# Nuclear membrane disruption in neurodegenerative diseases: Emerging perspectives

**DOI:** 10.4103/NRR.NRR-D-25-01127

**Published:** 2026-01-27

**Authors:** Shuo Yuan, Nicholas Essepian, Qingbo Wang, Lulu Jiang

**Affiliations:** 1 Department of Neuroscience, University of Virginia School of Medicine, Charlottesville, VA, USA; 2 Center for Brain Immunology and Glia (BIG), University of Virginia School of Medicine, Charlottesville, VA, USA

**Keywords:** Alzheimer’s disease, neurodegenerative disease, nuclear invagination, nuclear lamina, nuclear membrane disruption, nuclear pore complex, nucleocytoplasmic transport, nucleoporin

## Abstract

The nucleus, as the largest organelle within the cell, serves as the central hub for storing, replicating, and transcribing genetic information, thereby orchestrating vital cellular processes. In eukaryotic cells, the nuclear membrane is composed of several structural components: the outer and inner nuclear membranes, the nuclear pore complexes, and the underlying nuclear lamina, which together preserve the stability of the intracellular environment. Neurodegenerative disorders, such as Alzheimer’s disease and related dementias, are characterized by the gradual degeneration and loss of neuronal structure and function in the central nervous system. Growing evidence suggests that alterations in nuclear envelope architecture are closely associated with the onset and progression of these diseases. This article summarizes the information, focusing on the regulators of the cell nuclear membrane, as well as its pathophysiological processes and regulatory mechanisms in neurodegenerative diseases. Moreover, this paper discusses related research advances that provide novel insights into a deeper understanding of the nuclear membrane in disease progression and its potential as a therapeutic target.

## Introduction

Neurodegenerative diseases are among the leading causes of death and disability worldwide, affecting hundreds of millions of people. In 2021, they were responsible for millions of deaths globally, with both prevalence and absolute mortality increasing as populations age (Collaborators, 2024). Neurodegenerative diseases are slow-onset, long-lasting, and irreversible. Patients often exhibit varying degrees of cognitive impairment, such as impaired decision-making, learning difficulties, and memory loss (De Lucia et al., 2020; Gonzales et al., 2022; Weintraub et al., 2022). With the global population aging, the rising prevalence of neurodegenerative disorders, including Alzheimer’s disease (AD), Parkinson’s disease (PD), Huntington’s disease (HD), and amyotrophic lateral sclerosis (ALS), is exerting an increasing burden on societies worldwide (Guo et al., 2025). As disorders that cause neurological dysfunction by the progressive loss of neurons, neurodegenerative diseases are characterized by the main pathological manifestations of neuronal degeneration, necrosis, and accumulation of pathological hallmarks of protein aggregation in the brain and/or spinal cord, which are significant risk factors for dementia (Hou et al., 2019). The pathogenesis of neurodegenerative diseases is complicated and involves multiple pathological processes, including inflammation, oxidative stress, along with neuronal damage (Teleanu et al., 2022). Currently, neurodegenerative diseases can be categorized in three ways. The first is based on the main clinical features, such as PD, dementia, or motor neuron disease (Klineova and Lublin, 2018; Babazadeh et al., 2023). The second is according to the anatomical distribution of the neurodegenerative lesions, such as extrapyramidal disorders, spinal cerebellar degeneration, and frontotemporal lobar degeneration. The third one is categorized mainly by molecular abnormalities (Dugger and Dickson, 2017). The various neurodegenerative diseases and their main clinical manifestations are summarized in **Table 1**. Since it is difficult to regenerate and recover from nerve damage, related treatments are primarily focused on alleviating symptoms, and there is a lack of effective strategies to stop or significantly slow the onset and progression of neurodegenerative diseases, it is critical to discover the causative events and mechanisms involved in neurodegenerative diseases.

**Table 1:** Pathophysiology and clinical manifestations of neurodegenerative diseases

Diseases	Pathophysiology	Clinical performance	References
Alzheimer’s disease	Amyloid-β plaques; neurofibrillary tangles	Cognitive decline; memory loss	Scheltens et al., 2021
Amyotrophic lateral sclerosis	Motor nerve degeneration	Muscle weakness and atrophy; paresthesia	Feldman et al., 2022
Parkinson’s disease	Degenerative death of dopaminergic neurons; significant reduction in striatal dopamine content; formation of Lewy bodies	Characteristic motor symptoms; nonmotor symptoms	Kalia and Lang, 2015
Huntington’s disease	Huntington gene mutation in the short arm of chromosome 4, 4p16.3	Motor disorders; cognitive disorders; psychiatric disorders	Jimenez-Sanchez et al., 2017

In eukaryotic cells, the nucleus is the largest organelle and serves as the repository for genetic material in the form of DNA or chromatin. Essential cellular activities, including DNA replication and gene transcription, take place within this compartment. The nuclear membrane, which encloses the nucleus and separates it from the cytoplasm, safeguards nuclear integrity and genomic stability while enabling the spatial separation of replication, transcription, and translation processes. Structurally, it comprises the outer and inner nuclear membranes, the nuclear pore complexes, and the underlying nuclear lamina. At sites where the inner and outer membranes converge, large nuclear pore complexes, assembled from over 60 distinct proteins, facilitate the selective exchange of molecules between the nucleus and cytoplasm. Beneath the inner nuclear membrane lies a mesh-like structure called the nuclear lamina, with chromatin and the nucleolus located on its inner side (Pujadas Liwag et al., 2024). The nuclear lamina is primarily composed of type A and type B Lamin proteins (Wang et al., 2025). These proteins share structural features with cytoplasmic intermediate filaments and assemble into a stable nuclear framework through head-to-tail polymerization, which is critical for preserving nuclear envelope architecture and positioning nuclear pore complexes (Buxboim et al., 2023). The inner nuclear membrane contains numerous transmembrane proteins, including the lamin B receptor (LBR), Emerin, Lap2, SUN1, and Man1, which can bind to nuclear lamins or directly to chromatin or DNA, regulating processes such as chromatin assembly, DNA replication, and gene transcription (Marano and Holaska, 2025). The nuclear membrane, together with the nuclear pore complexes and the underlying lamina, forms the nuclear periphery that sustains a stable microenvironment for chromatin and nucleolar activities. This integrated structure mediates nucleocytoplasmic transport and is vital for cellular processes, such as proliferation, differentiation, development, and aging.

There is a close connection between changes in the peripheral structure of the cell nucleus and human diseases. It has been found that in tumor cells, the nuclear membrane structure is highly irregular. In senescent cells or tissues, the nuclear membrane structure is abnormal, heterochromatin disappears from the periphery of the nuclear membrane, and the structure of the nuclear lamina is altered. Advancing research has revealed that mutations in various nuclear membrane or membrane-associated proteins are closely linked to neurodegenerative disease (Lattanzi et al., 2011). Therefore, this review aims to provide comprehensive insights into the mechanisms of nuclear membrane disruption in neurodegenerative diseases and to elucidate how such alterations contribute to neuronal dysfunction and cell death.

## Literature Search

A detailed literature search was carried out across multiple bibliographic sources, including PubMed (via NCBI), Web of Science Core Collection, Embase (via Ovid), Scopus, and the Cochrane Library. Both primary research studies and review articles were considered to provide a comprehensive overview of existing evidence. The search strategy combined controlled vocabulary terms (such as MeSH and Emtree) with free-text keywords using Boolean operators AND/OR. The primary focus was on neurodegenerative disorders, specifically “Alzheimer’s disease,” “Parkinson’s disease,” “amyotrophic lateral sclerosis,” and “Huntington’s disease.” Additionally, terms related to nuclear envelope and lamina components, including “nuclear membrane,” “nuclear lamina,” “Lamin A/C,” “Lamin B,” and “LBR,” were included to capture studies addressing structural alterations in the nucleus. Keywords associated with pathogenic mechanisms, such as “tau,” “alpha-synuclein,” “protein aggregation,” “chromatin organization,” and “neurodegeneration,” were also incorporated.

To ensure studies specifically addressing nuclear membrane disruption were captured, titles and abstracts containing phrases such as “nuclear membrane disruption in neurodegenerative diseases” and “emerging perspectives” were screened. Searches were limited to English-language publications, without restriction on publication date, to encompass all relevant research. Furthermore, reference lists of included articles were manually examined to identify additional pertinent studies. Preprint servers, including bioRxiv and medRxiv, were also consulted to include the most recent findings. This structured approach allowed for a thorough and transparent identification of literature relevant to both neurodegenerative diseases and nuclear membrane dysfunction.

## Nuclear Membrane Disruption

**Figure 1:**
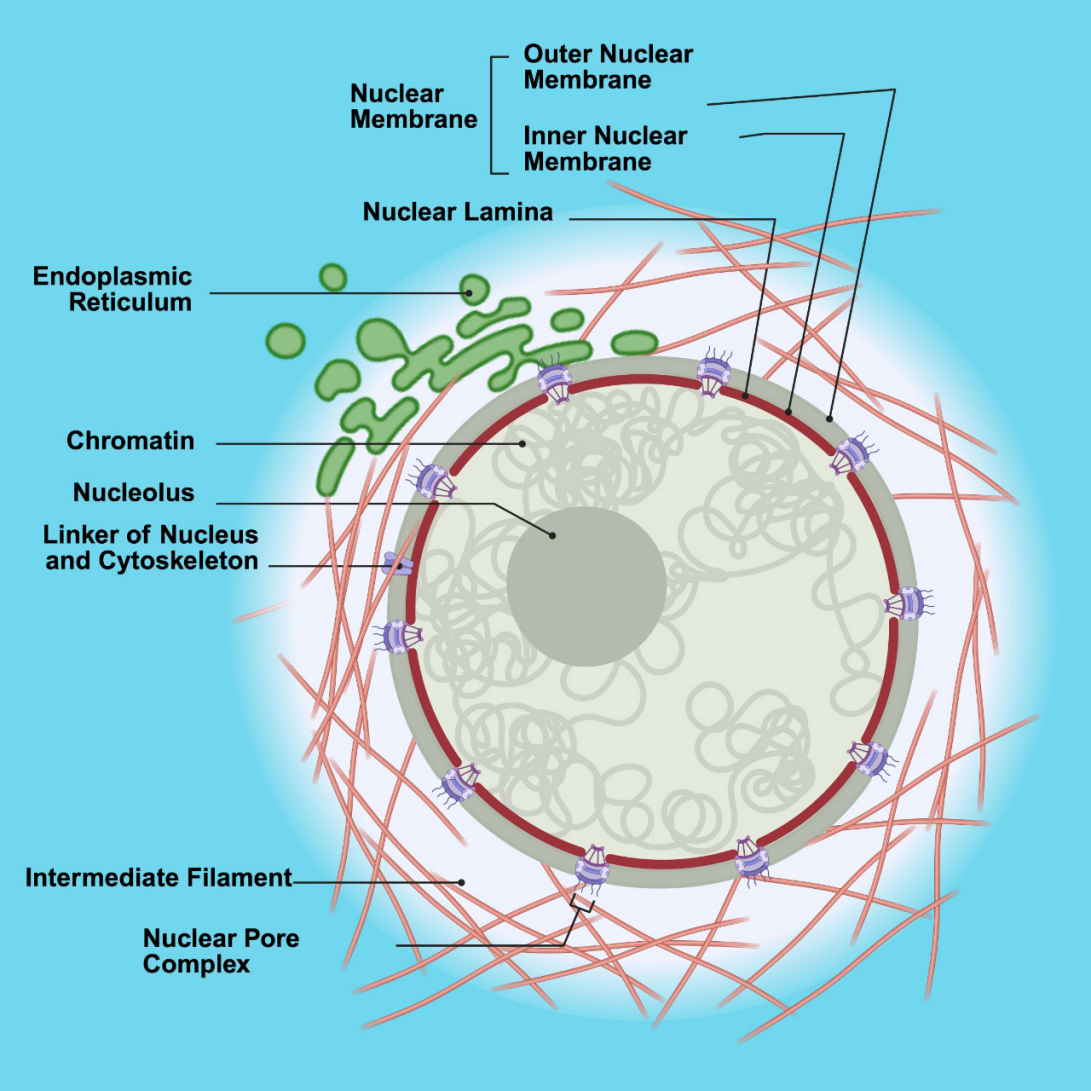
Structure of the nuclear membrane. The nuclear membrane is a double-layer membrane structure that wraps the nucleus of a eukaryotic cell. It is composed of an outer nuclear membrane (connected to the endoplasmic reticulum) and an inner nuclear membrane (attached to the nuclear laminin network). The surface of the nuclear membrane is embedded with nuclear pore complexes, which precisely regulate the selective transport of RNA, proteins, and other substances between the nucleus and the cytoplasm. The nuclear lamina on the inner side of the nucleus provides mechanical support for the nucleus and participates in chromatin anchoring. This structure not only protects the integrity of genetic material but also is a key interface for gene regulation. Its dysfunction is closely related to the occurrence of neurodegenerative diseases. Created in BioRender. Russo, L. (2025) https://BioRender.com/2u22ur2.

Eukaryotic cells possess a nucleus, where the nuclear membrane separates the nucleus from the cytoplasm, forming a natural barrier between the two (Ptak et al., 2024). As illustrated in **Figure 1**, the nuclear membrane is composed of an inner and an outer membrane. The outer membrane is continuous with the endoplasmic reticulum and contains numerous nuclear pores on its surface. These pores arise where the inner and outer membranes fuse to form circular openings that facilitate molecular exchange between the nucleus and the cytoplasm. Together with associated proteins, these openings constitute the nuclear pore complexes, which serve as critical gateways linking the two membranes and regulating the bidirectional transport of materials and signals across the nuclear boundary (Hachiya et al., 2021). Integral membrane proteins on the inner nuclear membrane are synthesized on ribosomes in the cytoplasm and subsequently transported into the nucleus (Shao and Hegde, 2011). This process requires specific signal sequences and is an energy-dependent active transport mechanism (Hachiya et al., 2021). The nuclear membrane is important in separating the nucleus and cytoplasm to maintain their respective functions without interference.

### Nuclear pore complex

The nuclear pore complex is a ring-like pore channel that communicates between the nucleoplasm and the cytoplasm, with a highly conserved structure. The nuclear pore complex contains three rings surrounding the central channel, which are loosely connected, with cytoplasmic fibrils attached to the cytoplasmic ring, nucleoplasmic fibrils attached to the nucleoplasmic ring, and a terminal ring at the base forming the nuclear basket (Schwartz, 2016). Nuclear pore complexes function as selective hydrophilic channels. Small molecules, such as ions and metabolites, pass through the nuclear pore complex via simple diffusion, whereas large molecules, including proteins, RNA, ribosomal subunits, and viral particles, require active transport mediated by nuclear translocation signals and transporter carriers (Li et al., 2016). Nuclear translocation signals include nuclear localization signals, which mediate inbound nuclear translocation, and nuclear export signals, which mediate outbound translocation. The precise regulation of molecules within the nuclear pore complex ensures its efficient nucleoplasmic translocation function (Atkinson et al., 2013; Knockenhauer and Schwartz, 2016). In addition to nucleocytoplasmic translocation, the nuclear pore complex is involved in DNA damage repair and mitosis. In proliferating cells, nucleoporins are involved in DNA damage repair by interacting with the DNA repair machinery to protect genome integrity (Sarangi and Zhao, 2015). In human cells, during mitosis, nucleoporins control the spatial positioning and activity of mitotic components by acting on the mitotic granules, stabilizing their interactions with microtubules, and ensuring the normal assembly of the spindle and accurate chromosome segregation (Forbes et al., 2015).

### Nuclear lamina

The primary function of the nuclear lamina is to maintain nuclear shape and regulate the spacing of nuclear pore complexes. The nuclear lamina is also involved in numerous cellular functions, including nuclear assembly, apoptosis, cell signaling, gene regulation, chromosome segregation, and DNA repair (Worman and Bonne, 2007). The nuclear lamina forms high-molecular-weight structures, in which type A and type B lamins interact to form a unique and interdependent fibrous meshwork. Lamin A interacts with multiple other structural proteins, including Emerin, Nup153, LAP2a, Nesprins, Actin, and 4.1R (Al-Haboubi et al., 2011), and forms a network with Emerin, 4.1R, and Actin at the nuclear membrane, enhancing nuclear stability (Zaremba-Czogalla et al., 2011). Lamins interact with transcription factors in regulating cell proliferation, differentiation, and apoptosis (Wilson and Berk, 2010). Both Lamin A and Lamin B possess DNA-binding sites that interact with chromatin in the nucleus, and changes in their quantity and structure impact the integrity of the chromosome set. *In vivo*, DNA labeling analyses of chromatin-lamin interactions have demonstrated that lamins dynamically interact with larger chromatin regions, known as nuclear lamina-associated structural domains. These domains are enriched with repressive histone marks, indicating that nuclear lamina-associated structural domains are unfavorable regions for chromatin aggregate localization. However, the specific role of lamins in the formation of nuclear lamina-associated structural domains remains unclear (Gesson et al., 2016). LBR, the Lamin B receptor, is an inner nuclear membrane protein with eight transmembrane domains. Its N-terminal region, comprising approximately 200 amino acids, is localized in the nucleus and can bind to heterochromatin protein 1, histones, and DNA (Nikolakaki et al., 2017). It has also been shown that LBR binds to importin-β to promote nuclear membrane assembly and is regulated by protein kinase phosphorylation (Nikolakaki et al., 2017). LBR deletion or mutation results in Pelger-Huët anomaly, although the underlying mechanism remains unknown (Young et al., 2021).

Changes in Lamin B1 expression levels are a hallmark of cellular senescence. Cellular senescence is a permanent state of cell cycle arrest triggered by various stresses, including sustained DNA damage, oxidative stress, and proto-oncogene activation. Upregulation or downregulation of Lamin B1 expression is associated with cellular senescence. Silencing Lamin B1 is accompanied by partial silencing of Lamin A, and replicative aging is associated with Lamin A oxidation, resulting in its functional inactivation (Dreesen et al., 2013). Moreover, the balance between Lamin B1 and Lamin A/C expression is a critical determinant of cellular aging. During senescence, reduced levels of Lamin B1 and its receptor LBR facilitate the detachment of heterochromatin from the nuclear lamina, thereby altering chromatin organization and gene expression profiles. These mechanical alterations extend to the nucleus; for instance, differentiation of embryonic stem cells is associated with enhanced chromatin compaction, resulting in increased nuclear stiffness (Politz et al., 2016).

### Linker of nucleus and cytoskeleton

The linker of nucleoskeleton and cytoskeleton (LINC) complex forms a continuous mechanical bridge spanning the inner and outer nuclear membranes. The LINC complex comprises two types of proteins: Nesprin (nuclear envelope spectrin repeat protein) and SUN (Sad1-UNC84 homology), both of which contain the KASH (Klarsicht, ANC-1, and Syne homology) structural domain (Starr and Fridolfsson, 2010). The LINC complex physically connects the cytoskeleton to the nucleus, transmits extracellular mechanical stimuli through the cytoskeleton, and influences chromatin localization and gene expression. The LINC complex is essential in regulating nuclear morphology, nuclear migration, meiosis, DNA damage repair, as well as gene transcription and expression. Nesprins determine the structure and distribution of perinuclear actin and intermediate filaments, influencing overall cell shape and mechanical response (Arsenovic et al., 2016). The knockdown of Nesprin1 reduces the tension of nuclear-bound actin filaments (Sakamoto et al., 2017). The LINC complex couples nuclei to microtubules, promoting neurogenesis and neuronal migration. Inhibition of Nesprin2G and SUN2 in migrating cells significantly impedes nuclear movement and centrosome reorientation (Cain et al., 2018). The LINC complex also participates in the DNA damage response (Lambert, 2019). Damage to SUN1/2 results in excessive DNA injury, increased genomic instability, and impaired DNA repair. During the sustained stretch, Emerin relocates from the inner to the outer nuclear membrane, interacting with non-muscle myosin IIA to promote cytoplasmic actin polymerization. This leads to a decrease in nuclear actin levels, which is counteracted by nuclear G-actin binding to RNA polymerase II to enhance transcription (Wei et al., 2020). In the RhoA/SRF/Mkl1 signaling pathway, SUN proteins regulate Mkl1 activation by signaling to RhoA, although the roles of SUN1 and SUN2 in this process are inconsistent. Additionally, the LINC complex regulates gene expression by controlling microRNA synthesis. It has been found that the nucleoplasmic domains of the major isoforms of SUN1 can act as cofactors to initiate microRNA biogenesis in specific cell types (Wong et al., 2021).

### Nuclear membrane disruption mechanisms

Nuclear membrane disruption refers to the localized loss of nuclear membrane integrity, which is often associated with senescence, affecting cell proliferation, activity, and tissue function (Kalukula et al., 2022). Since the discovery in 1996 that mutations in Emerin lead to X-linked Emery-Dreifuss muscular dystrophy, extensive research has uncovered that defects in various nuclear membrane or associated binding proteins contribute to a broad spectrum of human disorders (de Leeuw et al., 2018). Nuclear membrane disruption in cells is typically associated with deletions or mutations in nuclear lamina proteins and perinuclear heterochromatin disruption (Nader et al., 2021). The mutations in nuclear membrane proteins alter the perinuclear structure, thereby affecting chromatin organization and associated signaling pathways. Extensive experimental evidence indicates that cells with mutated nuclear membrane proteins exhibit altered chromatin localization and significant changes in histone modifications linked to gene expression.

A study has also demonstrated that mutations in nuclear membrane proteins disrupt cellular autophagy and lead to abnormal protein complex accumulation (Ramos et al., 2012). The specific nuclear components can be selectively degraded through nuclear autophagy, garnering significant attention (Luo et al., 2016). Nucleophagy depends on specific autophagy receptors such as p62 and NDP52, which possess LC3-interacting regions that are recognized by LC3B on the cytosolic surface of the autophagosome membrane (Papandreou and Tavernarakis, 2019). Chromatin and the nuclear membrane serve as targets for nuclear autophagy. During nuclear autophagy, nuclear components protrude from the nucleus, forming micronuclei or small nuclei in the cytoplasm near the nuclear membrane for degradation. These micronuclei can be readily detected by DNA or histone markers. These nuclear-containing components of autophagosomes eventually fuse with lysosomes to form autophagosomes (Luo et al., 2016). This suggests that the nuclear membrane, beyond being a target of autophagy, may also participate in autophagosome membrane formation and play a critical role in nuclear autophagy.

## Neurodegenerative Diseases and Nuclear Membrane Disruption

Increasing evidence suggests that deletions, mutations, or aberrant expression of nuclear membrane proteins can lead to a range of human diseases, especially age-related developmental and degenerative disorders. Therefore, this review focuses on neurodegenerative diseases, highlighting the role of nuclear membrane structure in their pathogenesis and its relationship with these disorders, intending to advance diagnosis and future therapeutic interventions.

### Alzheimer’s disease

AD is a common neurodegenerative disorder characterized by the deposition of amyloid-β plaques in the brain, the formation of neurofibrillary tangles due to abnormal phosphorylation and aggregation of Tau proteins in neuronal cells, and neuronal damage and necrosis (Moonen et al., 2023). Clinical manifestations include memory loss, language deficits, cognitive decline, impaired visuospatial skills, and executive dysfunction, along with personality and behavioral changes that significantly impact daily lives and social functioning of patients (Penke et al., 2020). Before lecanemab and donanemab were approved by the US Food and Drug Administration in 2021 for the treatment of early-stage AD (Espay et al., 2024), the only five US Food and Drug Administration-approved medications for AD, donepezil, galantamine, memantine, rivastigmine, and tacrine, had been in clinical use for 30 to 40 years (Barlow et al., 2021). Although lecanemab and donanemab represent newer therapeutic options, their efficacy remains debated, and their long-term safety and effectiveness require further evaluation (Daly et al., 2024). The clinical use of these two drugs has sparked ongoing controversy among clinicians and researchers (Espay et al., 2024). Current anti-AD drugs can only slow the progression of symptoms, as they are unable to halt or reverse the disease process, and symptoms often recur after discontinuation. Treating AD remains a significant challenge in clinical practice.

AD pathology is complex and the underlying pathogenesis remains elusive (Zhang et al., 2019). As research into the neurobiological mechanisms of AD progresses, several pathogenic mechanisms have been proposed, including central cholinergic damage, amyloid-β toxicity, and tau protein dysfunction. In hippocampal neurons of AD patients, cytoplasmic aggregation of nucleus accumbens transporter proteins α and NTF2 indicates a potential connection between the nuclear pore complex and AD (Lee et al., 2006). Additionally, abnormal cytoplasmic aggregation of pathogenic proteins with β-sheet misfolding disrupts nucleoplasmic transport in (Woerner et al., 2016). Pathological Tau compromises the structural and functional integrity of the nuclear pore complex, underscoring a direct link between nuclear pore complex dysfunction and AD. Additionally, phosphorylated Tau can bind directly to the nuclear pore protein Nup98, facilitating its aggregation (Eftekharzadeh et al., 2019). Further analyses showed that Nup98 aggregates with Nup62, which contains phenylalanine-glycine motifs, in cytoplasmic neurofibrillary tangles, while Nup133, lacking phenylalanine-glycine motifs, does not interact with Nup98. The mislocalization of nuclear pore proteins disrupts the function of the nuclear pore complex, impairing nucleocytoplasmic transport and the permeability barrier in AD neurons (Spead et al., 2022). Beyond the role of the nuclear pore complex in nucleocytoplasmic transport, a study has shown that Nup153 levels influence the maintenance and differentiation of hippocampal neural stem cells in mice (Leone et al., 2019). In hippocampal neural stem cells from AD mice, Nup153 protein levels and its interaction with SOX2 were reduced by 50%. Restoring Nup153 expression improved hippocampal neural stem cell functions, including proliferation, migration, differentiation, and neuronal maturation (Leone et al., 2019). These findings suggest that reduced Nup153 levels may contribute to AD neuronal deficits by disrupting its regulatory interaction with SOX2 in gene expression.

Furthermore, Lamin B protein deficiency disrupts normal nuclear movement, a critical process in neuronal development. Lamin B1/2-deficient mouse embryonic neurons exhibit significant migration defects, resulting in abnormal cortical neuronal layering and neonatal death (Coffinier et al., 2010, 2011). The duplication of the *Lamin B1* gene causes autosomal dominant leukodystrophy, characterized by widespread myelin loss in the central nervous system. Autosomal dominant cerebral leukodystrophy was the first disease linked to Lamin B1 mutations, highlighting the role of Lamins in brain diseases (Padiath et al., 2006). While the nuclear lamins in nonpathological neurons is crucial to inner nuclear envelope structure and assists in chromatin organization, DNA replication, and mediation of the nucleocytoplasmic interface (Coffinier et al., 2011; Young et al., 2012; Noguchi et al., 2021), pathogenic tau aggregation in AD neurons decreases nuclear tension (Sohn et al., 2023), induces structural changes in the Lamin B nucleoskeleton (Frost et al., 2016; Montalbano et al., 2019) and recruits Lamin B components to tau oligomers (Jiang et al., 2021; Jiang and Wolozin, 2021). Thus, pathological Tau aggregates are likely to infiltrate the nuclear membrane, where they interact with and mislocalize nuclear lamin proteins, leading to nuclear deformations and reorganization of chromatin.

### Amyotrophic lateral sclerosis

ALS is a neurodegenerative disorder with a poor prognosis, marked by the degeneration of motor neurons, gradual weakening of limb and trunk muscles, loss of speech and swallowing functions, and ultimately respiratory failure leading to death (Keon et al., 2021). The median survival after symptom onset is approximately 3 years, though there is significant variability among individuals (Xie et al., 2023), with around 10% of patients surviving for 10 years or more (Traxinger et al., 2013), and there is significant heterogeneity even in patients with monogenic familial ALS. The limited treatments available for ALS have minimal effect on disease progression and survival (Writing and Edaravone, 2017). The etiology of ALS is not well understood, and the pathogenic mechanisms that have been identified are diverse and heterogeneous. Its potential pathogenesis might involve genetic factors, excitatory amino acid toxicity, oxidative damage, neurotrophic factor deficiencies, mitochondrial dysfunction, abnormal protein aggregation, and impaired axonal transport, all of which may interact and influence each other. Since the discovery of the first mutation in the gene encoding the classical protein superoxide dismutase 1 (Kalia et al., 2023), more than 20 proteins have been identified whose altered function, distribution, mislocalization, and aberrant aggregation all influence the course of ALS (Maurel et al., 2018). The genetic variants increase the susceptibility to ALS or alter its clinical phenotype (Taylor et al., 2016).

Notably, it has been reported that in *Danio rerio* (zebrafish) and *Drosophila melanogaster* models, ALS-causing Annexin A11 mutations induce early nuclear dysfunction on Lamin B2 mislocalization, consistent with nucleocytoplasmic translocation abnormalities preceding behavioral deficits in ALS pathophysiology (Marchica et al., 2025). The most prevalent genetic cause of ALS is the GGGGCC hexanucleotide repeat expansion (HRE) in the C9orf72 gene. While disruptions in the nuclear pore complex and nucleocytoplasmic transport have been considered primary pathogenic mechanisms in C9orf72-related ALS, physiological levels of the HRE do not appear to increase the occurrence of nuclear lamina invaginations. Additionally, the overall nuclear morphology and size remain unaffected by C9orf72 HRE. Interestingly, the incidence of Lamin B1 invagination rises with cellular aging, independent of the presence of the HRE (Coyne and Rothstein, 2021).

### Parkinson’s disease

PD ranks as the second most common neurodegenerative disorder worldwide, following Alzheimer’s disease. It primarily affects older adults and is characterized by the degeneration of dopaminergic neurons in the substantia nigra, leading to motor impairments such as bradykinesia, muscle rigidity, and postural instability. A defining feature of PD is the formation of Lewy bodies, which are neuronal inclusions composed of aggregated α-synuclein (Armstrong and Okun, 2020). PD can be categorized into familial and sporadic. In familial PD, the etiology of the disease is determined by mutations in specific genes, whereas in sporadic PD, the etiology may be related to mutations in several different genes or may be unrelated to genetic factors (Malpartida et al., 2021). According to genome-wide association studies, more than 70 genetic loci have been identified as associated with the risk of developing PD (Nalls et al., 2019). In the pathogenesis of PD, the most characterized mutant forms of proteins have been identified, such as the multifunctional α-synuclein-rich and leucine-rich repeat kinase 2 (LRRK2); Parkin and PTEN-induced kinase 1 associated with mitochondrial autophagy; Parkinsonism associated deglycase (PARK7/DJ-1) associated with antioxidant function; and glucosylceramidase beta 1 associated with lysosomes (Simon et al., 2020). The exact pathogenesis of PD remains elusive, though multiple factors, including α-synuclein aggregation, neuroinflammation, oxidative stress, mitochondrial dysfunction, ferroptosis, and gut microbiota dysregulation, have been implicated in the disease (Dong-Chen et al., 2023). Current PD treatment primarily relies on dopamine replacement therapy, which effectively alleviates clinical symptoms but fails to halt disease progression. Moreover, its efficacy diminishes over time, and adverse reactions become more frequent with prolonged use (Jankovic and Tan, 2020).

There is cell cycle arrest in astrocytes in PD, which in turn is associated with increased levels of cell cycle inhibitors, including elevated p16^INK4a^ and loss of Lamin B1 (Martin-Ruiz et al., 2020). Moreover, nuclear structure defects are also caused by the interaction of LRRK2 with the Lamin B1 and B2 protein complexes. The G2019S mutation stimulates *LRRK2* gene activity, which directly or indirectly promotes the phosphorylation of Lamin B (Simonova et al., 2018). It has been shown that LRRK2 translocated to the nucleus, where it interacts directly with Lamin A/C. The nuclear membrane is abnormal in both LRRK2 knockdown and G2019S mutant transgenic mice (Shani et al., 2019). Additionally, the LRRK2 mutation R1441C increased nuclear membrane invagination and reduced nuclear recycling. However, these mice did not exhibit PD-associated pathology or motor deficits, suggesting that abnormal nuclear structure may represent an early feature of LRRK2-induced neuronal loss in PD (Tsika et al., 2014). The PD associated E3 ubiquitin ligase Parkin has been reported to directly interact with nuclear pore complex components. In human neuroblastoma cells, Parkin-mediated ubiquitination of Nup358 targets it for degradation via the ubiquitin-proteasome system, suggesting that mutations in Parkin could impair Nup358 turnover (Um et al., 2006). Additionally, Cho et al. (2012) explored gene-environment interactions in early PD and revealed that Nup358 dysfunction may increase susceptibility to neurotoxic stress.

### Huntington’s disease

HD, previously known as Huntington’s Chore, is an autosomal dominant neurodegenerative disease characterized by massive death of medium spiny neurons in the striatum, in which patients exhibit progressive motor, cognitive, and behavioral disorders (Jurcau, 2022). HD mainly involves the caudate nucleus, the nucleus accumbens, and the cerebral cortex. HD originates from genetic mutations. Structural alterations in Huntingtin predispose the protein to aggregation and toxicity. Mutant Huntingtin expresses polyglutamine due to its repetitive CAG sequence (Saudou and Humbert, 2016). The aggregation and accumulation of mutant Huntingtin can interfere with normal cellular functions by sequestering other proteins, affecting protein localization (Tabrizi et al., 2022; Aviner et al., 2024). It is associated with a variety of biochemical response abnormalities as well as cytoarchitectural malformations and dysfunctions, such as decreased secretion of brain-derived neurotrophic factor, mitochondrial dysfunction, excitotoxicity, abnormal post-translational modification of proteins, and disorders of peripheral immune system, which in turn lead to the development of the clinical symptoms of HD in patients (Kim and Fung, 2014). The causative gene for HD, IT15, is located on chromosome 4p16.3, and the number of CAG trinucleotide repeats in exon 1 is closely associated with the development of the disease (Pengo and Squitieri, 2024). In normal individuals, the copy number of CAG ranges from 6 to 25. Individuals with a copy number between 27 and 35 do not show clinical symptoms of HD, but there is a certain chance that the abnormal gene will be passed on to their offspring (Jurcau, 2022). The disease invariably manifests when the number of CAG repeats exceeds 40, and individuals with 60 or more CAG repeats may develop symptoms before the age of 21, presenting as juvenile-onset HD (Schultz et al., 2023).

A proteomics study in 2001 found that many NPC proteins, particularly Nup62, were able to co-aggregate with polyglutamine, suggesting that HD mutations may be toxic by altering nuclear pore complex function (Suhr et al., 2001). Normally, translocation of huntingtin proteins is mediated through the interaction of their N termini with Tpr, a nuclear pore basket protein (Spead et al., 2022). Mutant huntingtin has increased phosphorylation at its N-terminal serine-16, which disrupts its translocation and leads to nuclear aggregation. Mutations in huntingtin also lead to aggregation of this protein with the nuclear pore complex and nucleocytoplasmic transport components. There is a potential role for nuclear pore complex and nucleocytoplasmic transport dysfunction in HD as the disease progresses as highlighted by the presence of Nup62, Nup88, RanGAP1, and Gle1 in these aggregates in mice (Coyne and Rothstein, 2022; Spead et al., 2022). Furthermore, neurons derived from induced pluripotent stem cells of HD patients exhibit mislocalization of RanGAP1 and Nup62, accompanied by a marked decrease in the nucleocytoplasmic ratio of endogenous Ran, indicating impaired active transport between the nucleus and cytoplasm in these cells (Grima et al., 2017). Highly expressed pathological Tau has been shown to impair nucleocytoplasmic transport in AD brain tissue (Eftekharzadeh et al., 2019), which was further confirmed recently (Paonessa et al., 2019), while altered nuclear integrity and nucleocytoplasmic transport have been well established in HD (Gasset-Rosa et al., 2017; Grima et al., 2017).

## Conclusion and Perspective

Neurodegenerative diseases comprise a group of chronic, progressive disorders primarily characterized by pathological protein aggregation and neuronal loss. Multiple factors contribute to their development, including insufficient support from neurons or glial cells, disrupted axonal signaling, excessive activation of glutamate receptors, elevated reactive oxygen species, reduced mitochondrial energy production, abnormal accumulation or insufficient clearance of misfolded proteins, inflammatory responses, production of disease-specific proteins, and other cellular dysfunctions. Although the etiology and location of these diseases are different, they all share a common feature, which is the irreversible accumulation of pathological protein aggregation. Extensive research has been conducted on the mechanisms and treatment of neurodegenerative diseases, yet no effective or well-established therapies exist to prevent or cure these conditions. The nucleus serves as the central hub for storing genetic material, facilitating DNA replication, and enabling gene transcription within the cell. The physical properties of the nucleus, such as morphology, structure, mechanical properties, and location, are regulated by multiple components in the cytoplasm and intranuclear structures. Over the past decade, analyses have been conducted on the role of the nucleus, a complex and sophisticated subcellular structure, in neurodegenerative diseases. Although altered nuclear membrane properties, defective mechanotransduction, and related downstream effects have been implicated in disease progression, the driving forces underlying nuclear membrane disruption and its causal role in neurodegeneration remain elusive. Future in-depth investigations are needed to elucidate how nuclear membrane components contribute to the pathogenesis of neurodegeneration and how they interact with pathogenic protein aggregates. Recent advances in super-resolution microscopy sub-cellular proximity labeling and next-generation sequencing technologies are now enabling a more refined understanding of these processes.

**Figure 2:**
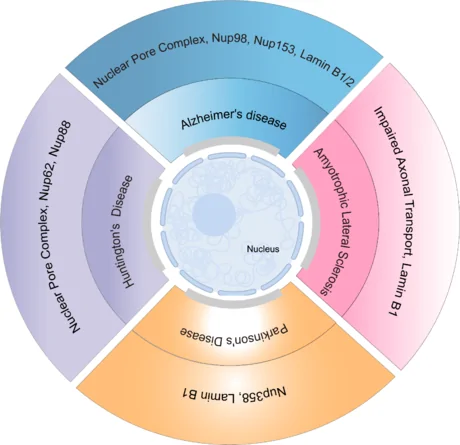
Nuclear membrane disruption and neurodegenerative disease. The nuclear membrane is a dynamic hub essential for neuronal health and survival. Its disruption, caused by genetic mutations, toxic aggregates, or cellular stress, leads to deleterious failures in nuclear transport, gene regulation, and DNA integrity. This cascade of dysfunction is increasingly recognized as a central driver in the pathogenesis of devastating neurodegenerative diseases such as Alzheimer’s disease, amyotrophic lateral sclerosis, Huntington’s disease, and Parkinson’s disease. Created in BioRender. Russo, L. (2025) https://BioRender.com/2u22ur2. Nups: Nucleoporins.

In this review, we summarize current analyses on nuclear membrane alterations and their regulatory roles in signal transduction during neurodegenerative diseases. The nuclear membrane undergoes characteristic changes in structure and properties that may influence cellular behavior by modulating gene expression within the nucleus (**Figure 2**). Beyond biochemical factors, multiple interconnected biophysical and molecular processes jointly shape the dynamic progression of neurodegeneration. However, major challenges remain, including the difficulty of recapitulating disease microenvironments *in vitro*, quantifying the contributions of individual factors to nuclear deformation, and dissecting disease-induced nuclear changes at the molecular level. Elucidating these mechanisms and targeting nuclear membrane dysregulation may ultimately offer new strategies for the diagnosis and treatment of neurodegenerative diseases.

## Data Availability

*Not applicable.*
